# Cross-cultural translation, adaptation, and validation of the Chinese versions of the athlete fear avoidance questionnaire and fear of return to sport scale

**DOI:** 10.1186/s13102-026-01808-4

**Published:** 2026-07-04

**Authors:** Siqi Liu, Young-Eun Noh, Almir Vieira Dibai-Filho, André Pontes-Silva, Tong Zhou, Yunxi Zhang

**Affiliations:** 1 School of Physical Education, Neijiang Normal University, Neijiang City, Sichuan Province 641100 China; 2 Faculty of Sports and Exercise Science, Universiti Malaya, Kuala Lumpur, 50603 Malaysia; 3 Postgraduate Program in Physical Education, Federal University of Maranhão, São Luis, MA Brazil; 4 Department of Physical Education, Wuhan Sports University, Wuhan, 430079 China; 5 Department of Physical Education, Chang’an University, Xi’an, Shaanxi Province 710064 China

**Keywords:** Sports injury rehabilitation, Fear avoidance, Return to sport, Psychological readiness, Scale validation, Injury recovery

## Abstract

**Background:**

Sports injuries not only impose periods of physical rest, but they can also provoke negative psychological responses, particularly fear, that can delay return to sport (RTS) and elevate reinjury risk. Although various instruments, including the Athlete Fear Avoidance Questionnaire (AFAQ) and the Fear of Return to Sport Scale (FRESS), are available to assess such fear-related factors, validated Simplified-Chinese versions have not yet been developed.

**Methods:**

This study conducted a systematic cross-cultural translation and psychometric validation of the AFAQ and the FRESS into Chinese (AFAQ-Ch and FRESS-Ch), following established international guidelines. Phase 1, which involved 30 athletes who had sustained an injury within the previous year (age: *M* = 20.6, *SD* = 2.4), comprised forward–backward translation, an expert panel review, and a face validity evaluation. Phase 2 assessed internal consistency, test–retest reliability, structural validity, and convergent validity among a larger sample of 204 Chinese injured athletes (age: *M* = 21.4, *SD* = 3.2), using the Simplified-Chinese Tampa Scale for Kinesiophobia (SC-TSK) and the Injury–Psychological Readiness to Return to Sport Scale (I-PRRS-Ch) as theoretically related comparison instruments.

**Results:**

Both the AFAQ-Ch and the FRESS-Ch demonstrated acceptable internal consistency (Cronbach’s α = 0.748 and 0.736, respectively) and acceptable test–retest reliability. The AFAQ-Ch showed good test–retest reliability, with an intraclass correlation coefficient (ICC) of 0.855 (95% CI [0.790, 0.900], F[96, 96] = 12.759, *p* < .001), whereas the FRESS-Ch demonstrated moderate test–retest reliability, with an ICC of 0.729 (95% CI [0.620, 0.810], F[96, 96] = 6.372, *p* < .001). Confirmatory factor analysis supported the structural validity of the AFAQ-Ch and FRESS-Ch, with acceptable model fit for the AFAQ-Ch and generally acceptable fit for the FRESS-Ch. Evidence of convergent validity was demonstrated through significant correlations. A significant moderate positive correlation was observed between the AFAQ-Ch and the FRESS-Ch (*r* = .484, *p* < .001).

**Conclusions:**

These findings indicate that the AFAQ-Ch and the FRESS-Ch can be reliably used in clinical and research settings to assess fear among Chinese-speaking athletes following injury.

**Supplementary Information:**

The online version contains supplementary material available at https://doi.org/10.1186/s13102-026-01808-4.

## Introduction

Sports injuries not only entail a period of physical rest [1], but they can also elicit negative emotional responses [2], including fear, anxiety, and depression [3, 4]. Among these negative emotions, fear has been widely examined because fear of reinjury may influence rehabilitation adherence, confidence in the injured body part, return to sport (RTS) readiness, and subsequent RTS outcomes [5–9]. For example, Kunnen et al. [5] conducted interviews with athletes who had undergone anterior cruciate ligament (ACL) reconstruction surgery following injuries sustained while playing soccer, finding that fear of reinjury was one of the primary psychological barriers faced by soccer players when returning to sport after injury. Additionally, using the Tampa Scale for Kinesiophobia (TSK [10]), Badiei et al. [11] conducted a cross-sectional survey of current and former athletes, discovering that fear related to sports injuries could influence athletes’ pain vigilance and pain memory, potentially contributing to the development of kinesiophobia. Moreover, using the Athlete Fear Avoidance Questionnaire (AFAQ), researchers found that fear avoidance was associated with injured athletes’ knee function [12], emotional state [13], and mental toughness [14], as well as with the timeliness with which they were able to return to their sports [15].

A recent qualitative study has suggested that the fear experienced after an injury is primarily the result of psychological factors rather than physical limitations [16]. A systematic review examining RTS outcomes after ACL reconstruction reported that, despite high rates of successful knee function recovery, the proportion of athletes returning to competitive sport remained relatively low [17]. This discrepancy also suggests that factors beyond physical recovery, particularly psychological factors, may play a critical role in RTS outcomes. Although athletes may achieve full physical recovery and receive medical clearance to RTS, they often continue to experience significant psychological challenges, particularly heightened fear [7–9]. Such fear can negatively influence their perceived functional performance [15, 18–20] and increase their risk of reinjury during the RTS phase [21]. These psychological barriers may ultimately delay the RTS process or, in some cases, prevent athletes from returning to sport [22–24].

In addition, emerging evidence suggests that psychological and physical recovery are not always synchronous [25]. For example, fear of reinjury may persist even when clinical indicators suggest satisfactory physical recovery. Conversely, incomplete physical recovery, residual pain, or perceived functional limitations may also contribute to fear and hesitation when considering RTS. Thus, psychological and physical recovery should not be viewed as separate processes, but as interacting dimensions of rehabilitation. Mental states may influence physical outcomes through several pathways, including reduced rehabilitation adherence, avoidance of sport-specific movements, guarded movement patterns, lower confidence in the injured body part, and delayed exposure to progressive loading [26]. These responses may limit functional recovery and potentially increase the risk of unsuccessful RTS or reinjury. For example, recent systematic review evidence has shown that psychological readiness measures are not only associated with subjective perceptions but may also predict recovery-related outcomes, including RTS success, physical function, and reinjury risk [26]. These findings highlight the important role of psychological factors in the rehabilitation process while also acknowledging that fear of RTS may partly reflect unresolved physical impairments.

In the sports context, the Anterior Cruciate Ligament–Return to Sport After Injury (ACL-RSI) Scale is widely used to assess athletes’ psychological readiness to RTS following injury [27]. Research has indicated that the ACL-RSI is more effective than the TSK [10] in identifying injury-related fear during the RTS process [28]. However, a key limitation is the ACL-RSI’s condition-specific design: It was developed for individuals recovering from ACL injuries and therefore cannot be applied to athletes returning from other types of injuries.

By contrast, the AFAQ and the Fear of Return to Sport Scale (FRESS) can be used to assess fear avoidance and fear of returning to sport, respectively, regardless of injury type [29, 30]. These instruments help address the above-mentioned limitation of the ACL-RSI by providing more generalizable measures of psychological responses to injury. As a result, both the AFAQ and the FRESS have been increasingly recommended and utilized in the field of sports medicine and psychology [1, 13–15, 26, 31]. Although both the AFAQ and the FRESS assess fear-related psychological responses following sports injury, they differ in their conceptual focus and temporal scope. The AFAQ is grounded in the fear-avoidance framework and captures broader pain-related fear and avoidance beliefs that may be present throughout the entire rehabilitation process [29]. In contrast, the FRESS focuses specifically on fear associated with RTS, particularly during the late rehabilitation phase [30]. At the same time, the two measures provide complementary perspectives on fear-related psychological responses rather than representing entirely redundant constructs. The AFAQ primarily assesses injury-related fear-avoidance beliefs, including pain-related concerns and avoidance tendencies during rehabilitation, whereas the FRESS focuses more specifically on fear-related responses associated with RTS decision-making. Conceptually, fear-avoidance beliefs during rehabilitation may shape athletes’ perceived vulnerability, confidence, and emotional readiness when approaching RTS. Therefore, the AFAQ and FRESS capture interconnected but non-identical aspects of fear-related psychological adaptation after sport injury.

This rationale is consistent with previous research in which related but distinct psychological responses after sport injury have been assessed within the same study framework. For example, O’Connor et al. [6] simultaneously employed the AFAQ and the Injury–Psychological Readiness to Return to Sport (I-PRRS) [32] to assess injury-related fear-avoidance beliefs and confidence in readiness to RTS among Irish collegiate athletes. Although the I-PRRS and FRESS assess different psychological dimensions, this previous work supports the view that fear-avoidance and RTS readiness are closely related but distinct components of post-injury psychological adaptation. Accordingly, validating both the AFAQ and FRESS within the same study framework may contribute to a more comprehensive assessment of fear-related psychological responses among injured athletes.

AFAQ is a 10-item unidimensional instrument developed to assess fear-avoidance beliefs among athletes recovering from sports-related injuries [29]. Respondents rate their agreement with each statement (e.g., “I am not sure what my injury is.”) on a 5-point Likert scale ranging from *not at all* (1) to *completely agree* (5). The total scores range from 10 to 50, with higher scores reflecting greater fear avoidance. The AFAQ has demonstrated good internal consistency (Cronbach’s *α* = 0.805) among Canadian university athletes. In addition, the AFAQ has been translated into German [33], Brazilian Portuguese [34], Arabic [35], and Italian [36] and validated in these contexts.

Notably, Item 2 of the AFAQ (“I am worried about my role with the team changing”) reflects a team-sport context, as it refers to potential changes in an athlete’s role within a team. Therefore, the relevance and interpretation of this item may differ when the scale is applied to individual-sport athletes. Athletes participating in individual sports may interpret the concept of “team role” differently depending on the structure and social context of their sport. However, the original AFAQ was developed and validated exclusively among team-sport athletes. Therefore, to minimize potential interpretive inconsistency and maintain conceptual clarity, only team-sport athletes were included in the present study.

The FRESS is a recently developed, unidimensional eight-item measure designed to assess athletes’ fear when returning to sport after injury [30]. Each item (e.g., “I feel insecure about returning to the sport”) is rated on a 5-point Likert scale ranging from *never* (0) to *always* (4). The total scores (0–100) are computed by summing item responses and multiplying the total by 3.125, with higher scores indicating greater fear related to the RTS process. Initial evidence from injured athletes in Brazil supports the scale’s test–retest reliability, with intraclass correlation coefficients (ICC) of 0.90, internal consistency (Cronbach’s *α* = 0.87), and acceptable model fit via confirmatory factor analysis (CFA). As a relatively new instrument, the FRESS has not been subjected to cross-cultural validation in any context outside that in which it was originally developed.

The injury rate among Chinese athletes remains high [37, 38]. Although the Chinese version of the Injury–Psychological Readiness to Return to Sport (I-PRRS-Ch) Scale is available to assess injured athletes’ confidence during the RTS process, psychological rehabilitation following sport injury is multidimensional and may also include fear-related responses [39]. However, this existing Chinese-language instrument primarily focuses on confidence-related aspects of RTS readiness, whereas validated Chinese-language tools specifically assessing fear and fear-avoidance responses remain limited. Consequently, reliance on a single Chinese-language instrument may not fully capture the broad spectrum of psychological responses experienced by injured Chinese athletes during rehabilitation and RTS decision-making, thereby limiting the identification of fear-related concerns that may interfere with successful RTS. For example, in an interview study involving 10 elite Chinese athletes recovering from injuries, Qin et al. [25] found that psychological rehabilitation lagged behind physical recovery, resulting in diminished performance upon RTS.

Given the central role of fear in postinjury recovery, there is a clear need for reliable and culturally appropriate measures to assess fear and fear avoidance among Chinese athletes. However, despite their relevance for understanding psychological readiness and RTS-related concerns, neither the AFAQ nor the FRESS has been validated for use in Chinese-speaking populations. This gap highlights the need for rigorous translation, cross-cultural adaptation, and psychometric validation to ensure that these measures are suitable for Chinese clinical and research settings. Therefore, this study aims to translate the AFAQ and the FRESS into Chinese (AFAQ-Ch and FRESS-Ch) and to evaluate their psychometric properties among Chinese athletes. Validated Chinese versions of these instruments will enable sports psychologists, clinicians, and coaches to more accurately identify fear-related barriers during rehabilitation and RTS decision-making, ultimately helping to reduce reinjury risk and support safer, more effective RTS pathways.

Participants in the present study included athletes ranging from low to high levels of sport participation, all with at least one year of training experience. The inclusion of lower-level athletes was intentional, as previous research suggests that athletes at lower competitive levels may be at a higher risk of injury due to relatively lower training intensity, physical preparedness, and competitive experience [40]. Therefore, broadening the inclusion criteria allowed us to capture a wider range of injury-related psychological experiences while also facilitating participant recruitment.

## Methods

### Study design

This study aimed to translate, culturally adapt, and psychometrically validate the AFAQ and the FRESS for use among Chinese-speaking populations. Ethical approval was obtained from the University of Malaya Research Ethics Committee (UM.TNC(P&I)/UMREC/3/5249). In addition, translation permissions were secured from the original developers of the AFAQ and the FRESS. We followed the consensus-based standards outlined in the COnsensus-based Standards for the selection of health Measurement INstruments (COSMIN) checklist [41, 42]. We divided this study into two phases. Phase 1 focused on the cross-cultural translation, content validity, and face validity of the AFAQ-Ch and the FRESS-Ch. Phase 2 aimed to assess the reliability (i.e., internal consistency and test–retest reliability), structural validity, and convergent validity of these two scales. Convergent validity was assessed by examining correlations between the target scales and theoretically related comparison instruments, including the Chinese version of the Tampa Scale for Kinesiophobia (SC-TSK) [43] and the I-PRRS-Ch [44]. Phase 1 data collection was conducted between January 2025 and March 2025, and Phase 2 data collection was conducted between May 2025 and July 2025. Phase 1 involved 30 participants (age: *M* = 20.6, *SD* = 2.4), while Phase 2 included 204 injured athletes (age: *M* = 21.4, *SD* = 3.2) for psychometric validation.

### Phase 1: cross-cultural translation of the original scales and assessment of the content validity and face validity of the AFAQ-Ch and the FRESS-Ch

**Fig. 1:**
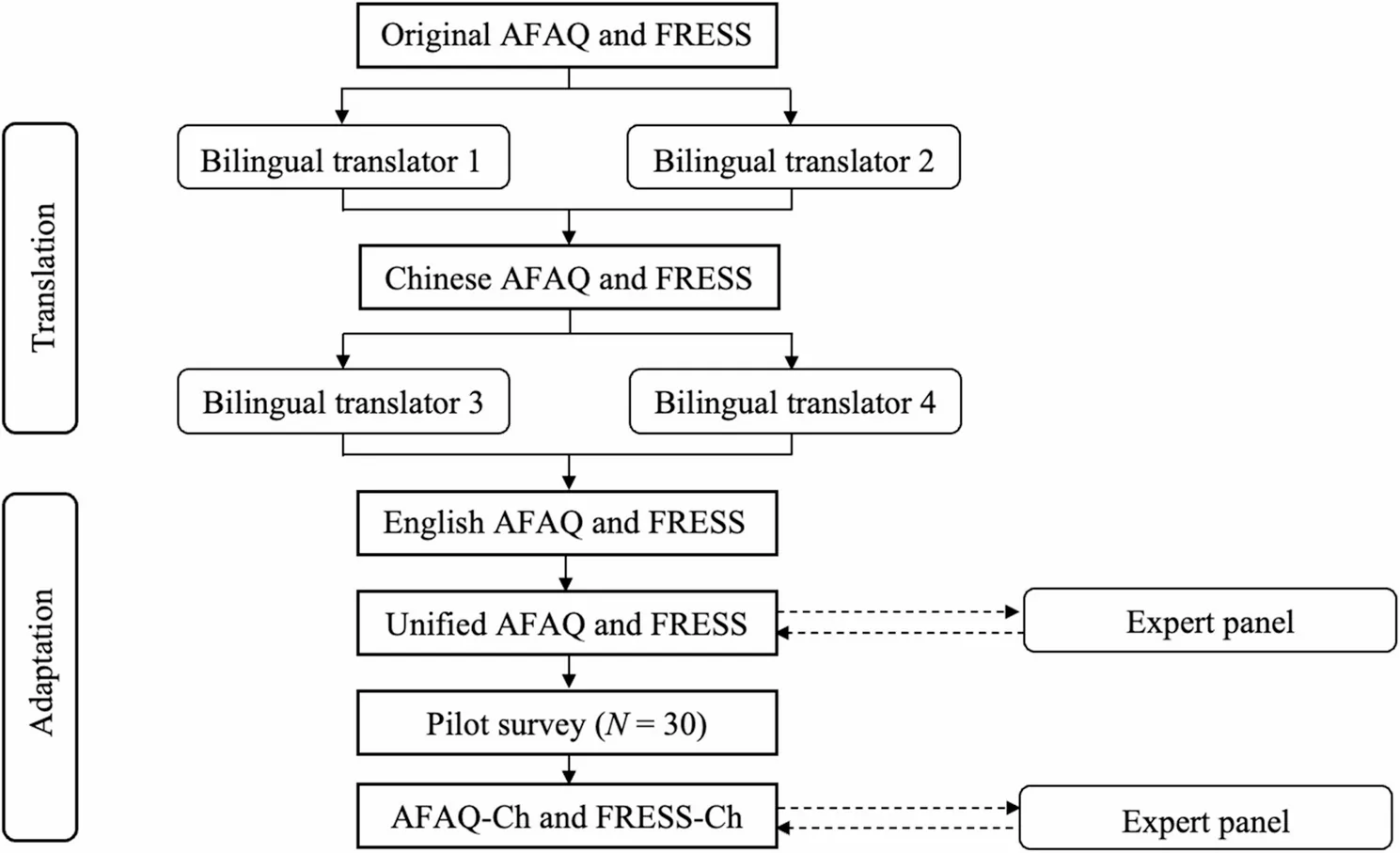
Flowchart of the Translation and Adaptation Procedures for the AFAQ and the FRESS. Note. FRESS = Fear of Return to Sport Scale; AFAQ = Athlete Fear Avoidance Questionnaire; FRESS-Ch = Chinese version of the Fear of Return to Sport Scale; AFAQ-Ch = Chinese version of the Athlete Fear Avoidance Questionnaire

The primary objectives of Phase 1 were the translation and cross-cultural adaptation of the AFAQ and the FRESS in accordance with the forward–backward translation method [45]. This process involved forward and backward translation by bilingual experts, followed by a review to assess content validity, which was conducted by an expert panel. A pilot study was subsequently carried out to evaluate face validity and to identify any potential issues related to translation and cultural adaptation. Figure 1 illustrates the flowchart of the translation and adaptation processes for the AFAQ and the FRESS.

### Translation

We invited four interpreters to translate the AFAQ and the FRESS into the AFAQ-Ch and the FRESS-Ch, using the forward–backward translation method [45]. Two English-speaking translators independently completed the forward translation into Chinese. Both forward translators were native Chinese speakers with sport-related expertise and 5 to 13 years of professional translation experience. They had experience in translation work for major international sporting events, including the Winter Olympic Games and the World University Games, as well as prior involvement in the translation and adaptation of international psychological instruments. Subsequently, two independent translators, who were blinded to the original English scales, conducted the backward translation into English. Under the coordination of the first author, the four translators compared the original English versions, the forward-translated Chinese versions, and the back-translated English versions to evaluate semantic accuracy and conceptual equivalence. Any discrepancies were discussed and resolved through consensus before the final Chinese versions were established. The preliminary forward translations were used only as working drafts for comparison and reconciliation and were not administered to participants.

### Content validity: a panel of experts

We invited three experts to assess the content validity of the AFAQ-Ch and the FRESS-Ch. All three experts were native Chinese speakers and used Simplified Chinese as their primary working language. Two of them were researchers with doctoral degrees who had published papers related to psychological rehabilitation from sports injuries, while one was a certified psychotherapist with eight years of relevant professional experience. After obtaining their consent, the first author sent them the AFAQ-Ch and the FRESS-Ch via email, requesting their professional insights and evaluations of the scales. Content validity was assessed using the content validity index (CVI) approach [46]. The CVI was calculated at both the item level (I-CVI) and the scale level (S-CVI), based on the experts’ ratings of the Chinese versions of the instruments.

### Inclusion and exclusion criteria of participants for assessing face validity

Participants were invited to take part if they met the following inclusion criteria: (a) age of 18 years or older; (b) engagement in team sports, as Item 2 of the AFAQ specifically addresses concerns related to team roles (e.g., “I am worried about my role with the team changing.”). Although discussions with the original scale developer suggested that athletes involved in individual sports could interpret the concept of “team role” according to their own sport context, the original AFAQ was developed and validated exclusively among team-sport athletes, including soccer, rugby, football, basketball, and hockey athletes. Therefore, the psychometric applicability of the scale among individual-sport athletes has not yet been established. In addition, potential differences in the interpretation of Item 2 between team-sport and individual-sport athletes remain unclear. To minimize potential interpretive inconsistency and maintain conceptual clarity, only team-sport athletes were included in the present study; (c) experience of a sports injury in the past year that led to the cessation of training or competition for more than 7 days, as Kalatakis-dos-Santos et al. [30] specified this period in their inclusion criteria for the scale; (d) more than 1 year of training or competition experience; (e) a training or competition frequency of at least three times per week before injury. The demographic characteristics of the pretest participants are presented in Table 1.

**Table 1:** Pretest Participants’ Characteristics (*N* = 30)

Demographics	Values	Percentage
Age	18–20	46.7
	21–23	43.3
	≥ 24	10
Training frequency (per week)	3–4 times	23.3
	5–6 times	70
	≥ 7 times	6.7
Types of sports participation	Basketball	26.7
	Volleyball	26.7
	Football	46.7
Sports career	1–2 year(s)	20
	2–3 years	50
	≥ 4 years	30

### Procedure

Participants in the pilot study were recruited online from sports clubs in Gansu, China, through the first author’s personal network. The first author contacted eligible individuals via WeChat, a multipurpose social media app that is one of the most widely used in China, and explained the purpose and procedures of this study before the data collection. Subsequently, participants indicated their consent by clicking a consent form before beginning the questionnaires. Participants used Wenjuanxing, a popular online survey platform in China, to complete the AFAQ-Ch and the FRESS-Ch. They also provided demographic information, including their gender, age, weekly training frequency, sports history, and types of sports practiced. Additionally, a screening question was included to determine whether they had experienced a sports injury in the past year that had resulted in the cessation of training or competition for more than 7 days. After completing the electronic questionnaire, the first author interviewed these participants via WeChat to assess the face validity of both the AFAQ-Ch and the FRESS-Ch. This included evaluating the clarity and comprehensibility of the questionnaire instructions, the wording of the items, and the response formats (e.g., how responses were indicated, such as selecting, ticking, or circling an option).

### Phase 2: assessment of the reliability and convergent validity of the AFAQ-Ch and the FRESS-Ch

The objective of Phase 2 was to validate the AFAQ-Ch and the FRESS-Ch in terms of their reliability and validity in a larger sample. We chose the SC-TSK [43] and the I-PRRS-Ch [44] to assess the convergent validity of the AFAQ-Ch and the FRESS-Ch. Both the I-PRRS and the TSK are widely used instruments for assessing psychological readiness related to RTS following injury [26, 39]. Moreover, the TSK was employed in the validation of the German version of the AFAQ [33]. Liu et al. [44] similarly used the SC-TSK to evaluate the convergent validity of the I-PRRS-Ch.

Giangreco et al. [33] reported a moderate positive correlation coefficient (*r* = .57, 95% CI [0.37, 0.71], *p* < .01) between the AFAQ (German version) and the TSK (German version) among injured athletes in Germany. Similarly, a longitudinal study conducted by Tito et al. [15], which involved 59 university athletes, found a significant moderate positive correlation between the AFAQ and the TSK (*r* = .308, *p* < .05). In addition, O’Connor et al. [6] surveyed 163 athletes in the postinjury RTS phase, finding a significant moderate negative correlation (*r* = − .59, *p* < .001) between the I-PRRS and the AFAQ.

Based on the findings of these three studies, we hypothesized that the AFAQ-Ch would be moderately positively correlated with the SC-TSK and moderately negatively correlated with the I-PRRS-Ch. While the relationship between the AFAQ and the FRESS has not yet been explored in the literature, given the moderate positive correlation reported between the AFAQ and the TSK [33], and considering that both the TSK and the FRESS assess fear-related responses following injury [10, 30], we further hypothesized a moderate positive correlation between the AFAQ-Ch and the FRESS-Ch. Additionally, for the FRESS-Ch, since there are no instruments with similar structures currently used in China, we hypothesized that the FRESS-Ch would be moderately positively correlated with the SC-TSK and moderately negatively correlated with the I-PRRS-Ch.

### Participants

#### Sample size

We calculated the sample size based on the COSMIN recommendation of seven times the number of items in the questionnaire, provided that this number is not below 100 [47]. The inclusion criteria for this phase were largely the same as those used in the pilot study, with one key difference. In this phase, participants were required to have sustained a sports injury that resulted in a minimum of 7 days’ absence from training or competition. The exclusion criteria included participants who did not wish to RTS or to regain their preinjury performance level, as well as those with cognitive impairments.

Participants’ characteristics for the first and second surveys are presented in Table 2.

**Table 2:** Participants’ Characteristics for the First (*N* = 204) and Second (*N* = 97) Surveys

Demographics	*N* = 204 (1st Survey)		*N* = 97 (2nd Survey)	
	Values	Percentage	Values	Percentage
Age	18–20	45.1	18–20	49.5
	21–23	27.9	21–23	27.8
	≥ 24	27	≥ 24	22.7
Training frequency (per week)	3–4 times	13.2	3–4 times	15.5
	5–6 times	50	5–6 times	49.5
	≥ 7 times	36.8	≥ 7 times	35.1
Types of sports participation	Basketball	26	Basketball	23.7
	Volleyball	27.5	Volleyball	23.7
	Football	46.6	Football	52.6
Site of injury	Knee	10.8	Knee	9.3
	Ankle	25	Ankle	27.8
	Lower back	25.5	Lower back	26.8
	Leg	22.1	Leg	21.6
	Arm	10.3	Arm	8.2
	Others	6.4	Others	6.2
Sports career (years)	1–3 year(s)	45.1	1–3 year(s)	45.4
	4–7 years	28.9	4–7 years	29.9
	7–9 years	10.8	7–9 years	12.4
	≥ 10 years	15.2	≥ 10 years	12.4

### Measurements

#### Demographic questionnaire

This questionnaire gathered demographic information about the participants, including their gender, age, training frequency per week, sports history, types of sports practiced, and current training/competition cessation time as a result of injury or cognitive impairments.

#### The Injury–Psychological Readiness to Return to Sport Scale (I-PRRS scale) [32]

The I-PRRS scale was developed to assess athletes’ confidence levels when returning to sport after an injury. The unidimensional I-PRRS scale has six items, each of which is rated on a scale from 0 (*no confidence at all*) to 10 (*complete confidence*). The total score (ranging from 0 to 60) is obtained by summing the scores for each item. A higher score indicates a greater confidence level in returning to sport. This study employed the I-PRRS-Ch [44], which has demonstrated good internal reliability (Cronbach’s *α* = 0.85) and excellent test–retest reliability (ICC = 0.98). The scale has also demonstrated good validity among injured Chinese athletes [44]. Its construct validity was supported by an average variance extracted (AVE) of 0.57, while its convergent validity was evidenced by a moderate negative correlation (*r* = − .42) between the I-PRRS-Ch and the SC-TSK.

#### The Tampa Scale for Kinesiophobia (TSK) [10]

The TSK was developed to measure fear of movement/(re)injury. This study used the SC-TSK [43], which has demonstrated strong psychometric properties among Chinese athlete populations and has previously been used to establish the convergent validity of the I-PRRS-Ch [44]. It consists of 11 items, comprising three dimensions: somatic focus, activity avoidance, and avoidance belief. Participants were asked to rate their level of fear or concern regarding injury on a 4-point Likert scale ranging from 1 (*strongly disagree*) to 4 (*strongly agree*). The total SC-TSK score (ranging from 11 to 44) reflects the extent of kinesiophobia, with a higher score indicating a higher level of fear. The reliability of the SC-TSK has been established as adequately high (Cronbach’s *α* = 0.883), with good test–retest reliability (ICC = 0.798) [43].

This phase utilized the Chinese versions of the AFAQ and the FRESS (AFAQ-Ch and FRESS-Ch), which were translated and culturally adapted during Phase 1.

### Procedure

Participants were recruited offline (including five sports clubs, 15 rehabilitation centers, and three hospitals). We explained this study’s purpose, participation criteria, and procedures to intermediaries (including coaches, physical rehabilitation therapists, and physiotherapists) via WeChat calls. When they expressed a willingness to assist, they were requested to distribute consent forms and paper questionnaires to potential participants who met the participation criteria. Each participant engaged voluntarily and was required to sign the provided consent form before completing the questionnaires. Subsequently, the intermediaries collected completed questionnaires from participants. To further assess the test–retest reliability of the AFAQ-Ch and the FRESS-Ch, we invited 100 participants who took part in the first survey, as recommended by Terwee et al. [48]. These participants were requested to complete the AFAQ-Ch and the FRESS-Ch again within 2 to 3 weeks of the initial administration [47]. This flexible time frame helped alleviate undue pressure on participants. In the second survey, a screening question (“Have you returned to sports?”) was included to ensure that athletes who had resumed sports activities were excluded from this study. Additionally, participants who had undergone surgery during this period were excluded. Following the completion of both surveys, the results of the questionnaires were used for the data analysis.

### Data analysis

#### Data screening

Prior to the data analysis, data screening was conducted during the coding of the paper-based questionnaires. Questionnaires with missing responses or those that did not meet the inclusion criteria were identified and excluded at this stage. After removing such cases, the remaining valid dataset was used for the statistical analysis.

#### Statistical analysis

We utilized IBM SPSS Statistics (Version 27) to examine floor and ceiling effects, convergent validity, internal consistency reliability, and test–retest reliability. A floor or ceiling effect is typically considered present if approximately 15% of respondents score at the lowest or highest possible level [48]. Convergent validity was examined using Pearson’s correlation coefficient (*r*) to assess the strength of associations among the AFAQ-Ch, FRESS-Ch, I-PRRS-Ch, and SC-TSK. Because Pearson’s *r* represents both the correlation coefficient and an effect size estimate, the magnitude of associations was interpreted as follows: coefficients exceeding 0.6 were categorized as high, values between 0.3 and 0.6 were classified as moderate, and values below 0.3 were considered low [49]. Before conducting Pearson correlation analyses, assumptions of approximate normality and linearity were examined using descriptive statistics, Shapiro–Wilk tests, and scatterplots. Although several variables demonstrated minor deviations from normality based on Shapiro–Wilk tests, the distributions and linear relationships were considered acceptable for Pearson correlation analyses given the sample size and the robustness of Pearson’s correlation to mild non-normality.

Given the substantial imbalance between male and female participants in Phase 2, gender-based subgroup analyses were not conducted. The female subsample was relatively small compared with the male subsample, which may have resulted in unstable estimates and limited statistical sensitivity for detecting gender-related differences. Therefore, the present analyses focused on the overall sample.

We calculated internal consistency by using Cronbach’s α to identify whether there were redundant or heterogeneous items in the questionnaires. We considered a value to be adequate based on a Cronbach’s α of greater than 0.70 [50]. In addition, corrected item–total correlations (CITC) and Cronbach’s α if-item- deleted were examined to further evaluate item-level internal consistency.

The test–retest reliability for the AFAQ-Ch and the FRESS-Ch was assessed using the ICC based on a two-way mixed-effects model for single measures (ICC [3, 1]). This approach accounted for both the random variability among participants and the fixed nature of the measurement methods. According to the criteria proposed by Koo and Li [51], ICC values below 0.50 indicate poor reliability, values of 0.50–0.75 moderate reliability, values of 0.75–0.90 good reliability, and values above 0.90 excellent reliability. We calculated the standard error of measurement (SEM) and the minimal detectable change (MDC) by using the total variance between the first and second measurements and the ICC [52]. The MDC is a linear transformation of the SEM, and it helps determine whether a change in score is meaningful or attributable to error [42].

CFA was conducted using IBM SPSS Amos 27.0 to examine the structural validity of the AFAQ-Ch and the FRESS-Ch. Based on the original scale development studies, both instruments were specified as unidimensional models. Model fit was evaluated using the comparative fit index (CFI), Tucker–Lewis index (TLI), root mean square error of approximation (RMSEA), standardized root mean square residual (SRMR), and the chi-square/degrees of freedom ratio (CMIN/DF).

**Fig. 2:**
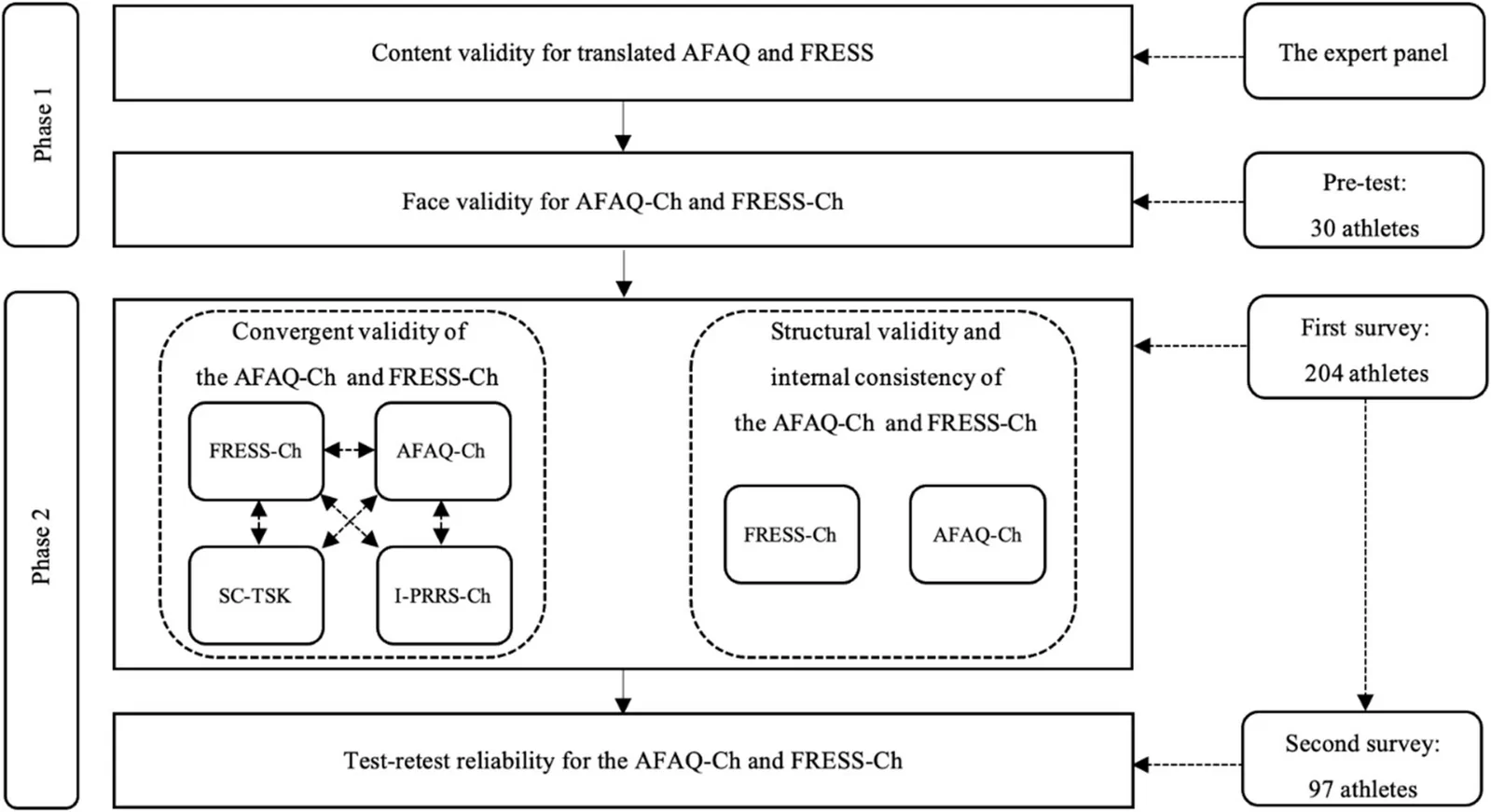
A Flowchart Outlining the Validity and Reliability Process. Note. FRESS = Fear of Return to Sport Scale; AFAQ = Athlete Fear Avoidance Questionnaire; FRESS-Ch = Chinese version of the Fear of Return to Sport Scale; AFAQ-Ch = Chinese version of the Athlete Fear Avoidance Questionnaire. I-PRRS-Ch = the Chinese Version of Injury-Psychological Readiness to Return to Sport scale; SC-TSK = the Chinese version of the Tampa Scale for Kinesiophobia

For the CFI and TLI, values below 0.90 were interpreted as indicating poor fit, values between 0.90 and 0.95 as acceptable fit, and values of 0.95 or above as good fit [53]. For the RMSEA, values of 0.05 or lower were interpreted as indicating good fit, whereas values between 0.05 and 0.08 suggested reasonable fit [53]. The 90% confidence interval for the RMSEA was also reported [53]. SRMR values below 0.08 were considered acceptable, with values below 0.05 indicating a good fit [53]. A CMIN/DF ratio below 5 was considered indicative of acceptable model fit in structural equation modeling [53]. Standardized factor loadings of 0.30 or higher were considered acceptable, with values above 0.50 indicating practically significant item loadings [54]. The flowchart outlining the validity and reliability process is presented in Fig. 2.

## Results

### Phase 1

#### Content validity

Regarding translation and cross-cultural adaptation, the experts agreed that no adaptation or significant change was necessary to produce the AFAQ-Ch and the FRESS-Ch. At the scale level, both the AFAQ-Ch and the FRESS-Ch demonstrated excellent content validity, with S-CVI/Ave and S-CVI/UA values of 1.00, indicating complete agreement among the experts across all items. The results of the CVI analysis are presented in Table 3.

**Table 3:** CVI Results for the AFAQ-Ch and the FRESS-Ch

Scale	Item	Expert 1	Expert 2	Expert 3	I-CVI	S-CVI/Ave	S-CVI/UA
AFAQ-Ch	Q1	4	4	4	1	1	1
	Q2	4	4	3	1		
	Q3	4	4	4	1		
	Q4	4	4	4	1		
	Q5	4	4	4	1		
	Q6	4	3	4	1		
	Q7	3	4	4	1		
	Q8	4	4	4	1		
	Q9	4	4	4	1		
	Q10	3	4	4	1		
FRESS-Ch	Q1	4	4	4	1	1	1
	Q2	4	4	4	1		
	Q3	4	3	4	1		
	Q4	4	4	4	1		
	Q5	4	4	4	1		
	Q6	4	4	3	1		
	Q7	3	4	4	1		
	Q8	4	4	4	1		

*AFAQ-Ch*  Chinese version of the Athlete Fear Avoidance Questionnaire, *FRESS-Ch*  Chinese version of the Fear of Return to Sport Scale, *CVI*  Content Validity Index, Expert ratings were based on a 4-point relevance scale (1 = not relevant, 4 = highly relevant). *I-CVI*  item-level content validity index, *S-CVI/Ave*  scale-level content validity index based on the average method, *S-CVI/UA*  scale-level content validity index based on universal agreement

#### Face validity

In this phase, 30 athletes (male = 16, female = 14, age: *M* = 20.6, *SD* = 2.4) with a history of sports-related injuries were invited to complete the AFAQ-Ch and the FRESS-Ch. All participants reported that they fully understood the questionnaire instructions, the wording of the items, and the response formats.

### Phase 2

#### Participants’ characteristics

In the data cleaning process, five responses had missing answers for certain items, and three responses had incomplete demographic information. After excluding these data, a total of 204 valid responses were retained from the first survey (male: *n* = 161; female: *n* = 43; age: *M* = 21.4, *SD* = 3.2). These responses were used to examine the Pearson correlations and assess the convergent validity of the AFAQ-Ch, the FRESS-Ch, the I-PRRS-Ch, and the SC-TSK. For the assessment of test–retest reliability, 100 participants from the initial survey were invited to complete a second survey. Of these, 97 participants completed the follow-up assessment, with a mean test–retest interval of 16.07 days (*SD* = 2.15; range = 14–21 days). This sample size remained well above the commonly recommended minimum threshold of 50 participants for reliability analysis [48] and was therefore considered sufficient for ICC estimation. Their responses were used to calculate the ICC, the SEM, and the MDC.

### Ceiling and floor effects

We examined total-score floor and ceiling effects for both scales. For the AFAQ-Ch, no participant obtained the minimum possible score of 10 or the maximum possible score of 50, indicating that 0% of participants reached the total-score floor or ceiling. Similarly, for the FRESS-Ch, no participant obtained the minimum possible score of 0 or the maximum possible score of 100, indicating no total-score floor or ceiling effects.

### Convergent validity

As hypothesized, the AFAQ-Ch and the FRESS-Ch showed significant correlations with the related psychological constructs, supporting their convergent validity (see Table 4). A significant moderate positive correlation was also observed between the AFAQ-Ch and the FRESS-Ch (*r* = .484, *p* < .001). Specifically, for the AFAQ-Ch, significant moderate negative correlations were found with the I-PRRS-Ch (*r* = − .371, *p* < .001) and moderate positive correlations with the SC-TSK (*r* = .339, *p* < .001). For the FRESS-Ch, strong negative correlations were identified with the I-PRRS-Ch (*r* = − .602, *p* < .001) and strong positive correlations with the SC-TSK (*r* = .722, *p* < .001), exceeding the hypothesized moderate magnitude of association.

**Table 4:** Correlation Matrix of AFAQ-Ch, FRESS-Ch, I-PRRS-Ch, and SC-TSK

Scale	AFAQ-Ch	FRESS-Ch	I-PRRS-Ch	SC-TSK
AFAQ-Ch	1			
FRESS-Ch	0.48*	1		
I-PRRS-Ch	–0.37*	–0.60*	1	
SC-TSK	0.34*	0.72*	-	1

*FRESS-Ch*  Chinese version of the Fear of Return to Sport Scale, *AFAQ-Ch*  Chinese version of the Athlete Fear Avoidance Questionnaire, *SC-TSK*  Chinese version of the Tampa Scale for Kinesiophobia, *I-PRRS-Ch*  Chinese version of the Injury-Psychological Readiness to Return to Sport

*p < .001

### Structural validity

**Fig. 3:**
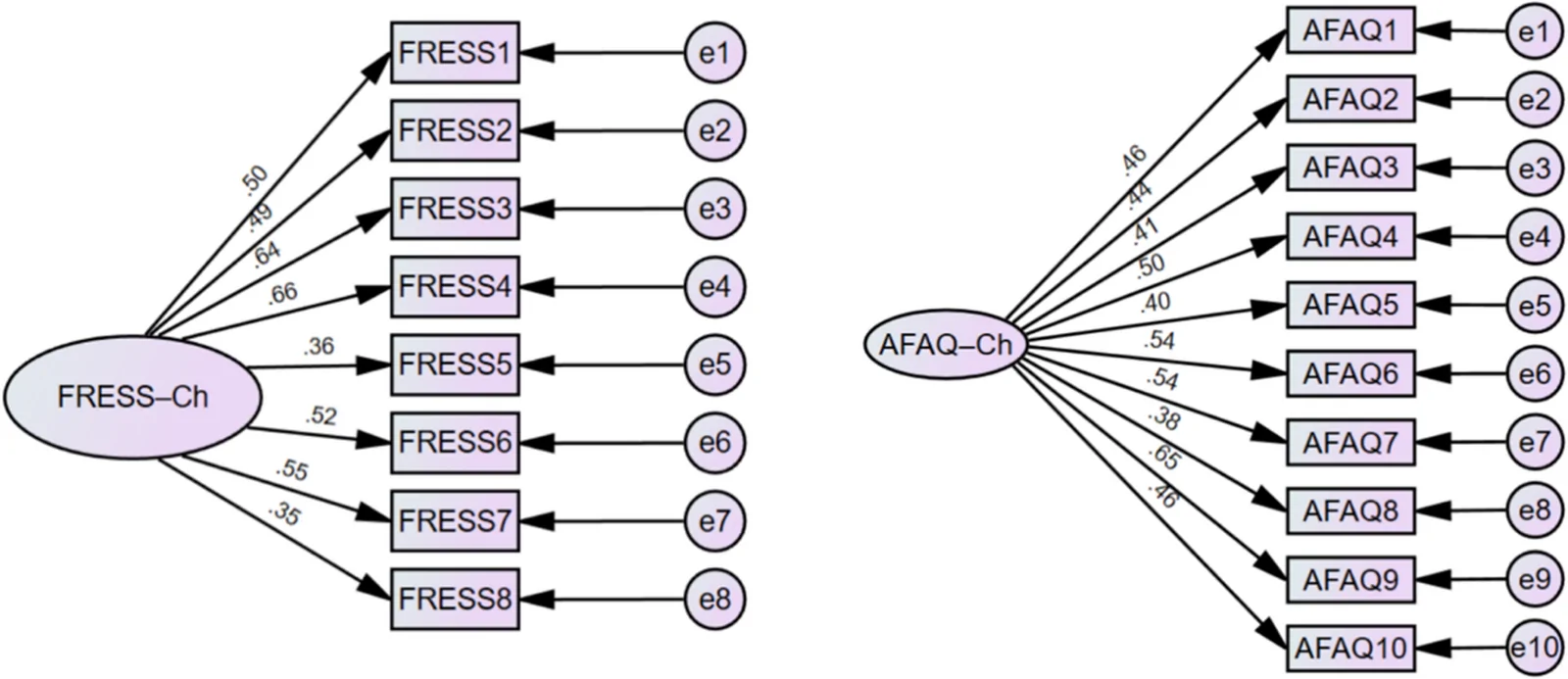
Confirmatory Factor Analysis Models of the FRESS-Ch and AFAQ-Ch. Note. FRESS-Ch = Chinese version of the Fear of Return to Sport Scale; AFAQ-Ch = Chinese version of the Athlete Fear Avoidance Questionnaire

The CFA results are presented in Table 5, and the standardized measurement models are illustrated in Fig. 3. Based on the interpretive guideline proposed by Tabachnick and Fidell (2007) [55], loadings around 0.32 may be considered poor but still interpretable, whereas loadings of 0.45, 0.55, 0.63, and 0.71 indicate fair, good, very good, and excellent loadings, respectively. In the present study, all standardized factor loadings exceeded the minimum interpretive threshold of 0.32. However, several items showed relatively low loadings, indicating that the strength of their association with the latent construct was modest. Therefore, although the CFA findings provided preliminary support for the unidimensional structures of the AFAQ-Ch and FRESS-Ch, these lower-loading items should be interpreted with caution and further examined in future validation studies.

**Table 5:** Confirmatory Factor Analysis Results for the AFAQ-Ch and FRESS-Ch

Scale	CMIN/DF	CFI	TLI	SRMR	RMSEA (90% CI)	Standardized Factor Loadings
AFAQ-Ch	1.313	0.958	0.946	0.049	0.039 [0.000, 0.068]	0.381–0.647
FRESS-Ch	2.034	0.917	0.883	0.057	0.071 [0.039, 0.103]	0.348–0.662

### Reliability and internal consistency

Both instruments demonstrated acceptable internal consistency, with Cronbach’s *α* coefficients of 0.748 for the AFAQ-Ch and 0.736 for the FRESS-Ch (see Table 6). The AFAQ-Ch demonstrated good test–retest reliability, with an ICC of 0.855 (95% CI [0.790, 0.900], *F*[96, 96] = 12.759, *p* < .001). The SEM was 2.10 and the MDC 5.82, indicating that changes of 5.82 points or more could be interpreted as true changes beyond measurement error. The FRESS-Ch showed moderate test–retest reliability (ICC = 0.729, 95% CI [0.620, 0.810], *F*[96, 96] = 6.372, *p* < .001), with an SEM of 5.32 and an MDC of 14.75, suggesting that score changes of greater than or equal to 14.75 points reflected real changes rather than measurement error. The CITC for the AFAQ-Ch items ranged from 0.342 to 0.536, indicating acceptable item homogeneity and supporting the stability of the scale’s internal structure. The CITC for the FRESS-Ch items ranged from 0.293 to 0.557. While one item (FRESS-Ch item 8) showed a slightly lower value (0.293), it was close to the recommended threshold of 0.30. In addition, the Cronbach’s α values if-item-deleted ranged from 0.707 to 0.737 for the AFAQ-Ch and from 0.681 to 0.732 for the FRESS-Ch, showing no substantial increase compared to the overall α coefficients. This suggests that no individual item significantly compromised the internal consistency of the scales. Overall, the results indicate acceptable item homogeneity and support the internal consistency of these scales.

**Table 6:** Reliability of the AFAQ-Ch and FRESS-Ch

Scale	Time 1 Mean ± SD (range)	Time 2 Mean ± SD (range)	ICC (95% CI)	SEM	MDC	Cronbach’s α	Interpretation
AFAQ-Ch	24.28 ± 5.46 (13–39)	26.21 ± 6.24 (14–41)	0.855 (0.790–0.900)	2.10	5.82	0.748	Good
FRESS-Ch	41.27 ± 15.22 (6.25–81.25)	55.67 ± 11.32 (34.375–81.25)	0.729 (0.620–0.810)	5.32	14.75	0.736	Moderate

*FRESS-Ch*  Chinese version of the Fear of Return to Sport Scale, *AFAQ-Ch*  Chinese version of the Athlete Fear Avoidance Questionnaire, *SEM*  Standard Error of Measurement, *ICC*  Intraclass Correlation Coefficient, *CI*  Confidence Interval, *MDC*  Minimum Detectable Change

## Discussion

This study aimed to translate, culturally adapt, and validate the Chinese versions of the AFAQ and the FRESS for use within a Chinese linguistic and cultural context. The findings indicate that both the AFAQ-Ch and the FRESS-Ch demonstrated acceptable reliability and generally supportive evidence of validity among injured Chinese athletes.

In this study, we found a significant moderate correlation between the AFAQ-Ch and the SC-TSK (*r* = .339, *p* < .001), consistent with the previous cross-cultural adaptation study of the German version of the AFAQ [33], which reported a significant moderate correlation between the AFAQ (German version) and the TSK (*r* = .57, *p* < .01). In addition, a longitudinal survey conducted by Tito et al. [15], which involved 59 university athletes, revealed a significant moderate correlation between the AFAQ and the TSK (*r* = .308, *p* < .05). Collectively, these findings suggest a meaningful relationship between kinesiophobia level and fear-avoidance beliefs among injured athletes. However, Tito et al. [15] noted that the TSK was not specifically designed for or worded toward athletic populations and therefore may not fully resonate with athletes. For instance, their survey found that the AFAQ was significantly associated with the time to RTS, whereas the TSK did not demonstrate a significant relationship with this variable, suggesting its limitations in capturing sport-specific fear following injury. Similarly, Meierbachtol et al. [28] argued that when measuring injury-related fears among injured athletes, using athlete-specific instruments (e.g., the ACL-RSI) was more effective than using the TSK. However, the ACL–RSI was specifically designed to assess psychological readiness during RTS among athletes with ACL injuries and therefore has limited applicability to athletes with other types of sports injuries. Therefore, we recommend the use of athlete-oriented psychological measures, such as the AFAQ, to assess fear levels following injury.

Although we initially hypothesized a moderate positive correlation between fear of returning to sport (FRESS-Ch) and kinesiophobia (SC-TSK), the observed correlation was substantially stronger than expected (*r* = .722), indicating a high degree of conceptual convergence between these constructs. As no prior studies have directly examined this relationship using comparable instruments, the hypothesis was necessarily exploratory and based primarily on theoretical reasoning and related evidence from adjacent constructs. From a measurement perspective, the high correlation may also be partially attributable to item-level overlap between the FRESS and the TSK. Specifically, several FRESS items conceptually overlap with SC-TSK items in terms of fear, vulnerability perceptions, and movement-related concerns. For example, FRESS item 1 (“I feel insecure about returning to the sport”) shares similarities with TSK item 1 (“I’m afraid that I might injure myself if I exercise”), as both reflect fear and insecurity related to physical activity following injury. Likewise, FRESS item 4 (“I am afraid to perform some movements with my body”) conceptually parallels TSK item 10 (“I can’t do all the things normal people do because it’s too easy for me to get injured”), as both capture fear of movement and perceived functional limitation. In addition, FRESS item 8 (“I think that my injury left my body vulnerable to further injuries”) overlaps conceptually with TSK item 5 (“My accident has put my body at risk for the rest of my life”) and TSK item 6 (“Pain always means I have injured my body”), as these items all reflect perceptions of bodily vulnerability and risk following injury. Despite this strong association, it is important to distinguish the functional focus of the two instruments. The FRESS-Ch is specifically designed to assess fear related to RTS, making it particularly relevant for RTS decision-making contexts. In contrast, the TSK primarily captures pain-related fear and movement avoidance. Therefore, the FRESS-Ch may provide more context-specific and clinically actionable insights into sport injury rehabilitation and RTS settings.

In addition, we found a significant moderate negative correlation between the AFAQ-Ch and the I-PRRS-Ch (*r* = − .371, *p* < .001), which is consistent with the findings of O’Connor et al. [6], who reported a moderate negative correlation (*r* = − .59, *p* < .001) between I-PRRS and AFAQ scores among Irish university athletes. These findings collectively suggest an inverse relationship between the confidence dimension of psychological readiness and fear-avoidance levels among injured athletes returning to sport.

Furthermore, a high negative correlation was found between the FRESS-Ch and the I-PRRS-Ch (*r* = − .602, *p* < .001), which may be attributed to the fact that both instruments assess psychological constructs directly related to the RTS phase, albeit from opposite perspectives—fear versus confidence. For instance, FRESS Item 1 (“I feel insecure about returning to the sport.”) and FRESS Item 6 (“I feel that I will not regain the confidence that I had before the injury.”) both reflect diminished psychological readiness and confidence. These contrast with I-PRRS Item 1 (“My overall confidence to play is … ”), which directly assesses an athlete’s general readiness to resume sports participation. Similarly, FRESS Item 2 (“I believe that my abilities in the sport will be compromised by my injury.”) focuses on perceived performance limitations, while I-PRRS Item 6 (“My confidence in my skill level/ability is … ”) evaluates the athlete’s perceived competence upon return, highlighting an inverse relationship in the perception of capability. Moreover, FRESS Item 4 (“I am afraid to perform some movements with my body.”) expresses fear regarding physical function, whereas the corresponding item in the I-PRRS (Item 5: “My confidence in the injured body part to handle demands of the situation is … ”) emphasizes self-assurance regarding the physical readiness of the injured body part. This pattern of conceptual mirroring between fear-based and confidence-based items likely contributed to the observed high negative correlation between the two measures. It further supports the convergent validity of the FRESS-Ch as a measure of fear of returning to sport.

Furthermore, our study identified a moderate correlation between the AFAQ-Ch and the FRESS-Ch (*r* = .484, *p* < .001), indicating a potential link between athletes’ fear levels and fear-avoidance beliefs during the RTS phase. This finding provides empirical support for the convergent use of these two measures in the present study. AFAQ-Ch captures injury-related fear-avoidance beliefs, whereas FRESS-Ch focuses more specifically on fear associated with return-to-sport decision-making. The observed correlation indicates that these constructs are related yet nonredundant, supporting the notion that injury-related fear and fear-avoidance beliefs represent complementary but distinct components of athletes’ psychological readiness for RTS.

In addition, the CFA findings provided generally support for the structural validity of both the AFAQ-Ch and FRESS-Ch. The AFAQ-Ch demonstrated good overall model fit, supporting the retention of its original unidimensional structure in the Chinese context. Similarly, the FRESS-Ch showed generally acceptable overall model fit, although the TLI value (0.883) was slightly below the conventional threshold of 0.90. This finding may reflect the sensitivity of the TLI to model complexity, sample characteristics, and the relatively small degrees of freedom in simple measurement models. Nevertheless, considering the overall pattern of fit indices, including the CFI, RMSEA, SRMR, and CMIN/DF, the results generally support the unidimensional structure of the FRESS-Ch among injured Chinese athletes. These findings are broadly consistent with the original development study of the FRESS, in which CFA supported a one-dimensional structure after five items with high error covariance were removed [30].

The original English version of the AFAQ reported a Cronbach’s *α* of 0.805 [29]. In our study, the Cronbach’s *α* for the AFAQ-Ch was 0.748. Comparable levels of internal consistency have been observed in other cultural adaptations of the AFAQ, including the German version (Cronbach’s *α* = 0.76 [33]), the Brazilian version (Cronbach’s *α* = 0.90 [34]), and the Arabic version (Cronbach’s *α* = 0.854 [35]). All these values exceed the recommended threshold of 0.70, supporting the AFAQ’s reliable internal consistency across different cultural contexts.

In addition, while the original English version of the AFAQ did not report test–retest reliability, our study yielded an ICC of 0.855 (95% CI [0.790, 0.900]), indicating good reliability. Similarly, high ICC values have been reported in other adaptations: the German version (ICC = 0.798; 95% CI [0.674, 0.876]), the Brazilian version (ICC = 0.85; 95% CI [0.71, 0.92]), the Arabic version (ICC = 0.885; 95% CI [0.784, 0.942]), and the Italian version (ICC = 0.95; 95% CI [0.93, 0.97]). All of these values exceed the accepted benchmark of 0.75, further supporting the AFAQ’s robust test–retest reliability across diverse cultural settings.

Finally, the Cronbach’s α for the FRESS-Ch in our study was 0.736, compared to 0.868 reported in the original Brazilian version among injured athletes. The ICC for the FRESS-Ch in our study was 0.729 (95% CI [0.620, 0.810]), indicating moderate reliability (between 0.50 and 0.75) but did not reach a good or excellent range. By contrast, the original version reported an ICC of 0.896 (95% CI [0.799, 0.948]). One possible explanation for the lower ICC observed in this study is the limited heterogeneity of the sample with respect to sports disciplines and age range. Because the original AFAQ includes role-related items framed within team-sport contexts, we restricted our sample to injured athletes from team sports (i.e., football, basketball, and volleyball) to minimize potential interpretive inconsistency and maintain conceptual clarity. Additionally, 73% of our participants were aged 18–23 years. These factors suggest that our sample had relatively low heterogeneity, which may have contributed to the ICC not reaching the good or excellent level.

Another possible explanation relates to differences in the test–retest interval. In the original study, reliability was assessed with a 7-day interval between assessments, whereas in the present study, a longer interval of 2 to 3 weeks was used. A longer retest interval may increase the likelihood of fluctuations in participants’ psychological states, particularly for dynamic constructs such as injury-related fear, thereby potentially reducing the stability of responses and leading to lower ICC estimates.

Importantly, the moderate test–retest reliability may also reflect the inherently dynamic nature of fear-related psychological responses during injury rehabilitation. Psychological states such as fear of reinjury, perceived physical vulnerability, confidence, and readiness to RTS may fluctuate as athletes progress through rehabilitation, experience symptom improvement, encounter setbacks, or approach RTS milestones. Therefore, changes in FRESS-Ch scores across the retest interval may not solely reflect measurement instability but may also represent genuine psychological change over time. This interpretation is consistent with recent literature suggesting that psychological readiness and injury-related fear should not be conceptualized as stable traits, but rather as evolving psychological states throughout the rehabilitation process [39]. In addition, no total-score floor or ceiling effects were observed for the FRESS-Ch, suggesting that the moderate ICC was unlikely to be explained by restricted score distributions. In clinical practice, this level of reliability implies that the scale can be used for group-level assessment and research purposes; however, caution should be exercised when interpreting individual-level changes, particularly in high-stakes RTS decision-making contexts.

In addition, FRESS-Ch item 8 (“I think that my injury left my body vulnerable to further injuries”) demonstrated a relatively lower corrected item–total correlation (CITC = 0.293), slightly below the commonly recommended threshold of 0.30. One possible explanation is that perceptions of bodily vulnerability following injury may vary substantially across athletes depending on injury severity, rehabilitation experience, sport type, and previous reinjury history. In addition, cultural differences in how athletes interpret and express concerns regarding physical vulnerability and reinjury risk may also have influenced responses to this item within the Chinese context. However, deleting this item did not substantially improve the overall Cronbach’s α coefficient, suggesting that the item did not meaningfully compromise the internal consistency of the scale. Therefore, the item was retained because it reflects a theoretically relevant aspect of fear of RTS and helps preserve consistency with the original scale structure. Nevertheless, future studies should further examine the wording, cultural interpretation, and structural contribution of this item in more diverse athletic populations.

### Limitations

This study has certain limitations. First, the sample exclusively consisted of young Chinese team-sport (i.e., football, basketball, and volleyball) athletes, with a mean age of 21.4 years and a predominance of male participants (78.9%). Because female athletes may experience injury-related fear, fear-avoidance beliefs, and return-to-sport concerns differently, future studies should recruit more gender-balanced samples and examine gender-based differences, measurement invariance, and potential differential item functioning across gender groups.

In addition, all data were collected within a single regional and cultural context in mainland China. These characteristics may result in limited sample representativeness, making it difficult to fully capture psychological differences among female athletes, athletes from different age groups, and those participating in individual sports. They may restrict the applicability of the findings across different cultural and linguistic contexts. For example, variations in written language (e.g., simplified Chinese used in mainland China versus traditional Chinese used in regions such as Taiwan) may influence the interpretation and applicability of the instruments. Therefore, the results of this study should be interpreted with caution when generalizing to broader and more diverse athletic populations.

Moreover, participants in Phase 1 were recruited through the first author’s personal network, while Phase 2 recruitment relied on intermediaries such as coaches and rehabilitation therapists. These recruitment approaches may have introduced selection bias, as athletes who were more willing to participate, more accessible through sport or rehabilitation networks, or more engaged in rehabilitation may have been overrepresented in the sample. Consequently, the psychometric estimates reported in this study may not fully reflect those of the broader population of injured athletes.

Second, content validity in this study was evaluated by a relatively small expert panel (*N* = 3). Notably, the S-CVI reached 1.00, indicating perfect agreement among experts. One possible explanation is the limited size of the expert panel, which may increase the likelihood of unanimous ratings. In addition, the high level of agreement may also reflect the strong conceptual clarity and item quality of the original scale. Therefore, while the findings support the adequacy of the translated items, future studies are encouraged to involve a larger and more diverse expert panel, ideally involving experts from sport psychology, sports medicine/ rehabilitation, psychometrics, and bilingual cross-cultural adaptation, to provide more robust evidence for item relevance, comprehensiveness, and comprehensibility.

Third, although CFA was conducted to examine the structural validity of the AFAQ-Ch and the FRESS-Ch, discriminant validity between the FRESS-Ch and conceptually related constructs, such as kinesiophobia measured by the SC-TSK, was not formally examined using additional structural validity approaches. Given the relatively high correlation observed between the FRESS-Ch and the SC-TSK, future studies are encouraged to further examine the factorial distinctiveness and discriminant validity of these constructs using larger samples and more comprehensive structural equation modeling techniques, such as CFA-based model comparisons, AVE-based comparisons, and heterotrait–monotrait ratio (HTMT) analyses.

Fourth, this study did not assess responsiveness or predictive validity. Because the present study used a cross-sectional design, it was not possible to determine whether the AFAQ-Ch and the FRESS-Ch are sensitive to changes over time or whether baseline scores can predict future rehabilitation outcomes, RTS readiness, or psychological rehabilitation. Future longitudinal studies should therefore examine the responsiveness of these instruments across the rehabilitation process and evaluate their predictive validity in relation to clinically and practically relevant outcomes, such as rehabilitation adherence, RTS timing, reinjury anxiety, and successful RTS.

Fifth, all measures relied on self-reported data, which may be subject to response bias. Injured athletes may underreport or overreport fear, fear-avoidance beliefs, or psychological readiness because of social desirability, perceived expectations from coaches or medical staff, or concerns that their responses could influence their RTS decisions. Such self-report bias may have affected the accuracy of the measured psychological constructs.

Sixth, although test–retest reliability was examined, stability estimates may be influenced by natural changes in athletes’ psychological states over time [39]. Injury-related fear, fear-avoidance beliefs, and psychological readiness are not static traits; instead, they may fluctuate as athletes progress through rehabilitation, experience setbacks, or approach RTS milestones. Such genuine psychological change may partially confound test–retest reliability estimates, highlighting the need for future studies to carefully select retest intervals and to interpret stability coefficients in light of the inherently dynamic nature of postinjury psychological responses. Alternatively, in line with recent recommendations [39], when evaluating psychological constructs, future researchers could consider using split-half reliability to assess internal consistency rather than relying on test–retest reliability.

Finally, the present study did not include athletes from certain high-risk sport types, such as combat sports or motorsports, and the sample was primarily composed of team-sport athletes. In addition, the study did not differentiate between specific injury types or include a sufficient representation of athletes with typical sport-specific injuries (e.g., ACL injuries in pivoting sports or shoulder injuries in overhead sports). Given that both injury mechanisms and psychological responses may vary across sport types and injury characteristics, this may limit the generalizability of the findings. Future research is encouraged to examine the applicability of the AFAQ-Ch and FRESS-Ch across a broader range of sports and injury profiles.

Importantly, because the present sample consisted exclusively of team-sport athletes, the psychometric properties and conceptual applicability of the AFAQ-Ch and FRESS-Ch among individual-sport athletes remain to be examined. In particular, concepts embedded in the AFAQ, such as “role with team” may carry different meanings in individual- versus team-sport environments, potentially leading to differences in item interpretation across sport types. Therefore, recommendations regarding the use of these scales among individual-sport athletes should be made with caution. Future research should recruit athletes from both individual and team sports and examine measurement invariance and cross-sport validity to determine whether the scales retain equivalent psychometric properties and conceptual meaning across different sporting contexts.

### Future research and implications

Since the AFAQ and the FRESS are relatively recently developed scales, we recommend that future studies establish clinically relevant cutoff scores to identify athletes at high risk of reinjury. Determining these thresholds will enhance the practical utility of these instruments in both research and clinical settings, enabling targeted psychological interventions to support injured athletes’ recovery and safe RTS. Although the present study did not establish formal clinical cutoff scores, the descriptive statistics, including mean scores and observed ranges, may provide preliminary reference values for future research and clinical interpretation among injured Chinese athletes. Future studies should employ larger and more diverse samples, longitudinal designs, and criterion-based approaches, such as receiver operating characteristic (ROC) analyses, to establish empirically supported cutoff values for identifying elevated fear-related psychological responses during rehabilitation and RTS.

Moreover, although certain psychological interventions, such as mindfulness interventions, have been shown to alleviate negative psychological responses among injured athletes [56], it remains unclear which interventions are most effective at different stages of injury recovery (acute phase, rehabilitation phase, RTS phase, etc.). Further research is also needed to determine what types of interventions are appropriate for athletes with varying levels of AFAQ and FRESS scores.

Clinical practitioners should incorporate psychological assessment into postoperative rehabilitation and RTS guidelines. We encourage Chinese practitioners to utilize the AFAQ-Ch and the FRESS-Ch to assess fear levels among injured Chinese athletes. Since fear following injury is modifiable, early identification and ongoing monitoring of these fears can facilitate the development of graded RTS plans. Such plans can address kinesiophobia and insufficient psychological readiness, thereby promoting rehabilitation progress and informed RTS decisions. These efforts have the potential to reduce the risk of reinjury related to psychological factors. It is also worth noting that although Item 2 of the AFAQ refers to team-related roles, the original developer indicated that this item can be interpreted more broadly to reflect an athlete’s perceived role or status, making it applicable to both team and individual sports. Future studies are encouraged to further examine its applicability across different sport types.

For coaches, these findings suggest the importance of monitoring movement quality and fatigue responses during sport-specific RTS drills. In addition to considering athletes’ self-reported confidence or fear, coaches may observe signs such as tremor, poor coordination, asymmetry, unstable landing or cutting mechanics, and deterioration in technique under fatigue. Where feasible, wearable sensors or mobile-based motion analysis tools can provide additional information about workload, movement control, and functional capacity. This may help coaches make more individualized RTS decisions and avoid progressing athletes based only on pain reduction or perceived readiness.

## Conclusion

The AFAQ-Ch and the FRESS-Ch demonstrated acceptable reliability and generally supportive evidence for validity, assessing injury-related fear and readiness for RTS among mainland Chinese athletes. These generally validated measures offer researchers and practitioners reliable tools for psychological assessment in injury rehabilitation and RTS contexts. They can effectively serve as screening tools to identify fear related to sports injuries among Chinese athletes following injury. However, given the sample characteristics—predominantly young, male, team-sport athletes from mainland China—these findings should be interpreted with caution and may not be generalizable to broader and more diverse athletic populations. Future research should examine their applicability across diverse sports, age groups, and cultural settings, as well as their predictive validity and responsiveness to change over time.

## Supplementary Information

Supplementary Material 1.

Supplementary Material 2.

## Data Availability

Data available on request from the authors. The data that support the findings of this study are available from the corresponding author upon reasonable request.
