# Supplementary material for: Cross-cultural translation, adaptation, and validation of the Chinese versions of the athlete fear avoidance questionnaire and fear of return to sport scale
Source: BMC Sports Sci Med Rehabil. 2026 Jul 4;18:449. doi: 10.1186/s13102-026-01808-4 (PMC13621716; doi:10.1186/s13102-026-01808-4)
Supplement: Supplementary file 1 [file 13102_2026_1808_MOESM1_ESM.docx]

# Fear of Return to Sport Scale (FRESS) – English version

During your time away from sports practice due to an injury, you have probably thought about your return to the sport. Therefore, how often in the past seven days have you had the thoughts or feeling listed below? Mark one option for each statement.

Response Options: 1 = Never | 2 = Hardly ever | 3 = Sometimes | 4 = Very often | 5 = Always

1. I feel insecure about returning to the sport.

2. I believe that my abilities in the sport will be compromised by my injury.

3. I feel that this injury can shorten my career in the sport.

4. I am afraid to perform some movements with my body.

5. I think that I should have spent more time in treatment to recover from the injury.

6. I feel that I will not regain the confidence that I had before the injury.

7. I am afraid of the criticisms and judgments of other people.

8. I think that my injury left my body vulnerable to further injuries.

# 重返运动恐惧量表– Chinese Version

在因伤暂停训练/比赛的这段时间里，你可能已经幻想过自己重新回到训练/赛场中的情形。请你回想过去七天内，以下这些想法或感受出现的频率。对于每一条陈述，请勾选一个最符合你想法或感受的选项。

作答选项：1 = 从不｜2 = 几乎从不｜3 = 有时｜4 = 经常｜5 = 总是

1. 我对重返运动感到不安。
2. 我认为这次受伤会削弱我的运动能力。
3. 我觉得这次受伤会缩短我的运动生涯。
4. 我害怕做出某些身体动作。
5. 为了更好的从伤病中恢复，我应该接受更长时间的治疗。
6. 我觉得自己的自信水平难以恢复到受伤以前。
7. 我害怕来自他人的批评和评价。
8. 我认为这次受伤让我的身体容易再次受伤。
