# Supplementary material for: Cross-cultural translation, adaptation, and validation of the Chinese versions of the athlete fear avoidance questionnaire and fear of return to sport scale
Source: BMC Sports Sci Med Rehabil. 2026 Jul 4;18:449. doi: 10.1186/s13102-026-01808-4 (PMC13621716; doi:10.1186/s13102-026-01808-4)
Supplement: Supplementary file 2 [file 13102_2026_1808_MOESM2_ESM.docx]

# Athlete Fear Avoidance Questionnaire (AFAQ) — English version

During the recovery process after a sports injury, athletes often experience varying levels of worry, avoidance behaviors, or fear of reinjury. The Athlete Fear Avoidance Questionnaire (AFAQ) is designed to assess your thoughts, emotions, and behavioral patterns related to injury over the past week. There are no “right” or “wrong” answers. Please answer each item based on your true feelings and experiences during the past seven days. Your responses will be used for research purposes only. All information will be kept strictly confidential. Read each statement carefully and select the option that best reflects your situation.

| **Rating** | **1** | **2** | **3** | **4** | **5** |
| --- | --- | --- | --- | --- | --- |
| **Meaning** | Not at all | To a slight degree | To a moderate degree | To a great degree | Completely agree |

| Statement | Rating |
| --- | --- |
| 1. I will never be able to play as I did before my injury |  |
| 2. I am worried about my role with the team changing |  |
| 3. I am worried about what other people will think of me if I don’t perform at the same level |  |
| 4. I am not sure what my injury is |  |
| 5. I believe that my current injury has jeopardized my future athletic  abilities |  |
| 6. I am not comfortable going back to play until l am100% |  |
| 7. People don’t understand how serious my injury is |  |
| 8. I don't know if I am ready to play |  |
| 9. I worry if I go back to play too soon I will make my injury worse |  |
| 10. When my pain is intense, I worry that my injury is a very serious one |  |

# 运动员害怕回避量表 — Chinese Version

在运动受伤后的恢复过程中，运动员常常会经历不同程度的担忧、回避行为或对再次受伤的恐惧。运动员害怕回避量表旨在评估您在过去一周中因受伤而产生的想法、情绪与行为模式。本问卷没有“对”或“错”的答案。请根据您在过去七天内的真实感受如实作答。您的回答将用于研究分析，所有信息将严格保密，仅用于科学研究目的。请阅读每一条陈述，并选择最符合您情况的选项。

| **评分** | **1** | **2** | **3** | **4** | **5** |
| --- | --- | --- | --- | --- | --- |
| **意义** | 完全没有 | 稍有 | 一般程度 | 很大程度 | 完全同意 |

| 陈述 | 评分 |
| --- | --- |
| 1. 我再也无法回到受伤前的运动状态了 |  |
| 2. 我担心我在运动队中的角色将被替代 |  |
| 3. 我担心如果我的运动表现没有达到受伤以前的水平，其他人会对我有意见 |  |
| 4. 我不确定我的伤势现在情况如何 |  |
| 5. 我认为我目前的伤情会损害我今后的运动能力 |  |
| 6. 在确认百分之百康复以后，我才能安心的重返训练/比赛 |  |
| 7. 大家都无法理解我这次受的伤有多严重 |  |
| 8. 我不确定我是否准备好了重返训练/比赛 |  |
| 9. 我担心如果我受伤后太快重返训练/比赛，我的伤情会变得更糟糕 |  |
| 10. 当我受伤部位的疼痛感非常强烈时，我担心我的受伤状况非常严重 |  |
